# 972. Hand Burns and Patient-reported Outcomes: A Comparative Analysis in Adults with Matched Burn Size

**DOI:** 10.1093/jbcr/irag033.569

**Published:** 2026-04-06

**Authors:** Sophie Hoffmann, Jeffrey C Schneider, Colleen M Ryan, Kaitlyn L Chacon, Huan Deng, Karen J Kowalske, Haig A Yenikomshian, Andrew Humbert

**Affiliations:** Spaulding Rehabilitation Hospital Boston, Thusis, Graubunden; Spaulding Rehabilitation Hospital, Mass General Brigham, Harvard Medical School, Boston, MA; Massachusetts General Hospital, Harvard Medical School, Boston, MA; Spaulding Rehabilitation Hospital, Boston, MA; Spaulding Rehabilitation Hospital, Harvard Medical School, Boston, MA; Department of Physical Medicine and Rehabilitation, University of Texas Southwest Medical Center, Dallas, TX; Division of Plastic and Reconstructive Surgery, Keck School of Medicine, University of Southern California, Los Angeles, California; University of Washington Department of Rehabilitation Medicine, Seattle, WA

## Abstract

**Introduction:**

Hand burns represent a small surface area but are associated with a significant and disproportionate functional impairment. Total Body Surface Area (TBSA) fails to capture the impact of hand involvement on recovery. Evidence on its influence on patient-reported outcomes remains limited. The study hypothesized that survivors with hand burns would demonstrate worse patient-reported outcomes than those matched by TBSA and sex and without hand burns.

**Methods:**

Adults aged 18 years or older enrolled in a national longitudinal database between 2015 and 2025 were included. Participants with hand burns were matched to controls without hand burns by TBSA and sex. Outcomes included PROMIS-29 domains of physical function, pain interference, fatigue, depression, anxiety, sleep disturbance, and social participation. Descriptive statistics compared the characteristics of the two groups. Regression models examined the association between hand burn status and PROMIS scores over time, controlling for demographic and clinical factors.

**Results:**

A total of 742 adult burn survivors (371 matched pairs) were included. Burn size for the two groups were similar (mean% [SD]: 8.0 [10.4] vs 8.2 [10.4]). Within the hand-burn cohort, 60% were unilateral; hand grafts were unilateral 53%, bilateral 16%, and none 31%; amputations included digits-only 5.1% and below-elbow 0.3%. In linear mixed-effects models, patients with hand burns had significantly higher physical function scores than those without (β = 2.1; 95% CI = 0.7, 3.5; *p*=.003). While the results were not statistically significant, there was a trend toward worse anxiety and sleep disturbance but better depression, fatigue, social participation, and pain interference in hand-burn survivors.

**Conclusions:**

When matched on burn size and demographics, patients with hand burns had significantly higher physical function scores compared to those without, while other PROMIS-29 outcomes did not differ significantly. The results of this study did not provide evidence of greater functional or social deficits based on cutaneokinematic burden of burn. The results are limited by the data collected in the data set. Further work is needed to understand the clinical importance of cutaneokinematic distribution of injury.

**Applicability of Research to Practice:**

A more granular analysis of the cohort's clinical characteristics (e.g., grafts, bilateral involvement, amputations) is warranted to elucidate the specific factors driving the observed outcomes. It will also guide the future rehabilitation efforts toward patients with hand burns.

**Funding for the study:**

This work was supported by the National Institute on Disability, Independent Living, and Rehabilitation Research.

